# Prime numbers in typical continued fraction expansions

**DOI:** 10.1007/s40574-023-00349-9

**Published:** 2023-02-28

**Authors:** Tanja I. Schindler, Roland Zweimüller

**Affiliations:** Fakultät für Mathematik, Universität Wien, Oskar-Morgenstern-Platz 1, 1090 Wien, Austria

**Keywords:** Continued fractions, Prime numbers, Stochastic limit theorems, Primary 11K50, 28D05, 37A25, 37C30, 37A50

## Abstract

We study, from the viewpoint of metrical number theory and (infinite) ergodic theory, the probabilistic laws governing the occurrence of prime numbers as digits in continued fraction expansions of real numbers.

## Introduction

Ever since Gauss [10] declared his interest in the intriguing statistical properties of sequences of *digits* ${\textsf{a}}_{n}(x)$, $n\ge 1$, in the *continued fraction* (*CF*) *expansion* of real numbers $x\in I:=(0,1]$,

$$\begin{aligned} x=\left[ {\textsf{a}}_{1}(x),{\textsf{a}}_{2}(x),\ldots \right] =\frac{1}{{\textsf{a}}_{1}(x)+\dfrac{1}{{\textsf{a}}_{2}(x)+\dfrac{1}{{\textsf{a}} _{3}(x)+\cdots }}} \end{aligned}$$

(and, in particular, mentioned that this led to questions he could not answer), the *metrical theory of continued fractions* has attracted the attention of many mathematicians. In the present paper we will be interested in the *prime digits* of *x*, i.e. those ${\textsf{a}}_{n}(x)$ which happen to belong to the set ${\mathbb {P}}$ of prime numbers. To single them out, we define, for $x\in I$ and $n\ge 1$,

$$\begin{aligned} {\textsf{a}}_{n}^{\prime }(x):=\mathbbm {1}_{{\mathbb {P}}}({\textsf{a}}_{n}(x))\cdot {\textsf{a}}_{n}(x)=\left\{ \begin{array}{ll} {\textsf{a}}_{n}(x) &{} \text {if }{\textsf{a}}_{n}(x)\in {\mathbb {P}}\text {,}\\ 0 &{} \text {otherwise.} \end{array} \right. \end{aligned}$$

(There is hardly any danger of misinterpreting this phonetically perfect symbol as a derivative.) The purpose of this note is to point out that it is in fact possible—with the aid of the prime number theorem and recent work in (infinite) ergodic theory and in the probability theory of dynamical systems—to derive a lot of information about the occurrences and values of prime digits in CF-expansions of (Lebesgue) typical numbers. Besides stating the theorems themselves it is also our aim to show some newer more general results in ergodic theory in action. While many analogous versions of the following statements have directly been proven for the continued fraction digits, today, it is possible to deduce them or the version for the prime digits from more general theorems.

The paper is structured as follows. In Sect. 2 we give results about the pointwise behaviour of the prime digits in the continued fraction expansion and in Sect. 3 about their distributional behaviour. Then we start with the proof sections, i.e. in Sect. 4 we state general results about the Gauss map and in Sects. 5 and 6 we give the proofs of the results from Sects. 2 and 3 respectively.

## Main results—pointwise matters

We first consider questions about the pointwise behaviour of the sequence $({\textsf{a}}_{n}^{\prime })_{n\ge 1}$ on *I*. Throughout, $\lambda$ denotes Lebesgue measure on (the Borel $\sigma$-field ${\mathcal {B}}_{I}$ of) *I*, and *almost everywhere *(*a.e.*) is meant w.r.t. $\lambda$. For the sake of completeness, we also include a few easy basic facts, e.g. that for a.e. $x\in I$, the proportion of those $k\in \{1,\ldots ,n\}$ for which ${\textsf{a}}_{k}(x)$ is prime converges:

### Proposition 2.1

(Asymptotic frequency of prime digits)We have

$$\begin{aligned} \lim _{n\rightarrow \infty }\,\frac{1}{n}\sum _{k=1}^{n}{\mathbbm {1}}_{{\mathbb {P}}} \circ {\textsf{a}}_{k}=\frac{1}{\log 2}\,\log \prod _{{\textrm{p}}\in {\mathbb {P}} }\left( 1+\frac{1}{{\textrm{p}}({\textrm{p}}+2)}\right) { \quad a.e.} \end{aligned}$$

The results to follow can best be understood (and proved) by regarding $({\textsf{a}}_{n}^{\prime })$ as a stationary sequence with respect to the Gauss measure (cf. §4 below). The first statement of the next theorem is parallel to the classical Borel-Bernstein theorem (cf. [5, 7]) the third and fourth statements are in accordance with [16]. As usual, *i.o.* is short for "infinitely often", i.e.  for "infinitely many indices". We denote the *iterated logarithms* by$\ \log _{1}:=\log$ and $\log _{m+1}:=\log \circ \log _{m}$, $m\ge 1$. Furthermore, we define the maximal entry ${\textsf{M}}_{n}^{\prime }:=\max _{1\le k\le n}{\textsf{a}}_{k}^{\prime }$, $n\ge 1$.

### Theorem 2.1

(Pointwise growth of prime digits and maxima)  

**a)** Assume that $(b_{n})_{n\ge 1}$ is a sequence in $(1,\infty )$. Then

2.1 $$\begin{aligned} \lambda (\{{\textsf{a}}_{n}^{\prime }>b_{n}\text { i.o.}\})=\left\{ \begin{array}{ll} 1 &{} \text {if }\sum _{n\ge 1}\frac{1}{b_{n}\log b_{n}}=\infty \text {,}\\ 0 &{} \text {otherwise.} \end{array} \right. \end{aligned}$$

**b)** Moreover, if $(b_{n})_{n\ge 1}$ is non-decreasing, then

2.2 $$\begin{aligned} \lambda (\{{\textsf{a}}_{n}^{\prime }>b_{n}\text { i.o.}\})=\lambda (\{{\textsf{M}} _{n}^{\prime }>b_{n}\text { i.o.}\})\text {. } \end{aligned}$$

**c)** Let $(c_n)_{n\ge 1}$ and $(d_n)_{n\ge 1}$ be sequences in $(1,\infty )$ with $d_n \rightarrow \infty$ and $c_n\le d_n^{0.475}$ for large *n*. Then

$$\begin{aligned} \lambda (\{d_n\le {\textsf{a}}_{n}^{\prime }\le d_n (1+1/c_n)\text { i.o.}\}) =\left\{ \begin{array}{ll} 1 &{} \text {if }\sum _{n\ge 1}\frac{1}{c_{n}d_n\log (d_n)}=\infty \text {.}\\ 0 &{} \text {otherwise.} \end{array} \right. \end{aligned}$$

**d)** Let $(d_n)_{n\ge 1}$ be a sequence of primes, then

$$\begin{aligned} \lambda (\{{\textsf{a}}_{n}^{\prime }= d_n \text { i.o.}\})=\left\{ \begin{array}{ll} 1 &{} \text {if }\sum _{n\ge 1}\frac{1}{d_n^2}=\infty \text {,}\\ 0 &{} \text {otherwise.} \end{array} \right. \end{aligned}$$

### Remark 2.1

The exponent 0.475 in c) comes from estimates for the error term in the prime number theorem and might be improved by future research.

### Example 2.1

A straightforward calculation shows that

$$\begin{aligned} \lambda (\{{\textsf{a}}_{n}^{\prime }>n\log _{2}^{\gamma }n\text { i.o.}\}) = \left\{ \begin{array}{ll} 1 &{} \text {if }\gamma \le 1\text {,}\\ 0 &{} \text {otherwise,} \end{array} \right. \end{aligned}$$

and this remains true if ${\textsf{a}}_{n}^{\prime }$ is replaced by ${\textsf{M}}_{n}^{\prime }$. We thus find that

$$\begin{aligned} \underset{n\rightarrow \infty }{{\overline{\lim }}}\,\frac{\log {\textsf{a}} _{n}^{\prime }-\log n}{\log _{3}n}=\,\underset{n\rightarrow \infty }{{\overline{\lim }}}\,\frac{\log {\textsf{M}}_{n}^{\prime }-\log n}{\log _{3} n}=1{ \quad a.e.} \end{aligned}$$

As a consequence of Theorem 2.1 b), observing that the series $\sum _{n\ge 1}1/(b_{n}\log b_{n})$ converges iff $\sum _{n\ge 1}1/(\rho \,b_{n}\log (\rho \,b_{n}))$ converges for every $\rho \in (0,\infty )$, we get

### Corollary 2.1

If $(b_{n})_{n\ge 1}$ is non-decreasing, then

2.3 $$\begin{aligned} \underset{n\rightarrow \infty }{{\overline{\lim }}}\,\frac{{\textsf{a}}_{n}^{\prime } }{b_{n}}=\,\underset{n\rightarrow \infty }{{\overline{\lim }}}\,\frac{{\textsf{M}}_{n}^{\prime }}{b_{n}} = \left\{ \begin{array}{ll} \infty \text { a.e.} &{} \text {if }\sum _{n\ge 1}\frac{1}{b_{n}\log b_{n}}=\infty \text {,}\\ 0 \text { a.e.} &{} \text {otherwise.} \end{array} \right. \end{aligned}$$

In particular,

2.4 $$\begin{aligned} \underset{n\rightarrow \infty }{{\overline{\lim }}}\, \frac{{\textsf{a}}_{n}^{\prime }}{n\,\log _{2}n}=\, \underset{n\rightarrow \infty }{{\overline{\lim }}}\, \frac{{\textsf{M}}_{n}^{\prime }}{n\,\log _{2}n} =\infty \text { a.e.} \end{aligned}$$

A convenient condition for the criterion above is provided by

### Lemma 2.1

Let $(b_{n})_{n\ge 1}$ be a sequence in $(1,\infty )$ for which $b_{n}/n$ increases. Then

$$\begin{aligned} \underset{n\rightarrow \infty }{{\overline{\lim }}}\,\frac{n\log _{2}n}{b_{n} }>0\text { implies }\sum _{n\ge 1}\frac{1}{b_{n}\log b_{n} }=\infty \text {.} \end{aligned}$$

As in the case of the full digit sequence $({\textsf{a}}_{n})_{n\ge 1}$, the peculiar properties of $({\textsf{a}}_{n}^{\prime })_{n\ge 1}$ are due to the fact that these functions are not integrable. A general fact for non-integrable non-negative stationary sequences is the non-existence of a non-trivial strong law of large numbers, made precise in a), c) and d) of the next result, where c) is in the spirit of [22]. However, it is sometimes possible to recover a meaningful limit by *trimming*, i.e. by removing maximal terms. In the case of $({\textsf{a}} _{n})_{n\ge 1}$, this was first pointed out in [8]. Assertion b) below gives the proper version for the $({\textsf{a}}_{n}^{\prime })_{n\ge 1}$.

### Theorem 2.2

(Strong laws of large numbers)  

**a)** The prime digits satisfy

$$\begin{aligned} \lim _{n\rightarrow \infty }\,\frac{1}{n}\sum _{k=1}^{n}{\textsf{a}}_{k}^{\prime }=\infty \text { a.e.} \end{aligned}$$

**b)** Subtracting ${\textsf{M}}_{n}^{\prime }$, we obtain a trimmed strong law,

2.5 $$\begin{aligned} \lim _{n\rightarrow \infty }\,\frac{\log 2}{n\,\log _{2}n}\left( \sum _{k=1} ^{n}{\textsf{a}}_{k}^{\prime }-{\textsf{M}}_{n}^{\prime }\right) =1\text { a.e.} \end{aligned}$$

**c)** For sequences $(b_{n})_{n\ge 1}$ in $(1,\infty )$ satisfying $b_{n}/n\nearrow \infty$ as $n\rightarrow \infty$,

2.6 $$\begin{aligned} \underset{n\rightarrow \infty }{{\overline{\lim }}}\,\frac{1}{b_{n}}\sum _{k=1} ^{n}{\textsf{a}}_{k}^{\prime }=\infty \text { a.e. iff }\sum _{n\ge 1}\frac{1}{b_{n}\log b_{n}}=\infty \text {,} \end{aligned}$$

while otherwise

2.7 $$\begin{aligned} \lim _{n\rightarrow \infty }\,\frac{1}{b_{n}}\sum _{k=1}^{n}{\textsf{a}}_{k} ^{\prime }=0\text { a.e.} \end{aligned}$$

**d) ** But, defining ${\overline{n}}(j):=e^{j\log ^{2}j}$, $j\ge 1$, and $d_{n}^{\prime }:={\overline{n}}(j)\,\log _{2}{\overline{n}}(j)/\log 2$ for $n\in ({\overline{n}}(j-1),{\overline{n}}(j)]$ gives a normalizing sequence for which

2.8 $$\begin{aligned} \underset{n\rightarrow \infty }{{\overline{\lim }}}\,\frac{1}{d_{n}^{\prime }} \sum _{k=1}^{n}{\textsf{a}}_{k}^{\prime }=1\text { a.e.} \end{aligned}$$

The trimmed law from b) shows that the bad pointwise behaviour described in c) is due to a few exceptionally large individual terms ${\textsf{a}}_{n}^{\prime }$ which, necessarily, have to be of the order of the preceding partial sum $\sum _{k=1}^{n-1}{\textsf{a}}_{k}^{\prime }$. In fact, almost surely, the partial sum will infinitely often be of strictly smaller order than the following term, see statement a) below. We can also ask whether, or to what extent, the terms from the thinner sequence $({\textsf{a}}_{n}^{\prime })_{n\ge 1}$ come close to the partial sums $(\sum _{k=0}^{n-1}{\textsf{a}}_{k})_{n\ge 1}$ of the unrestricted one. The answer is given by the dichotomy rule in statement b) of the next result.

We shall tacitly interpret real sequences $(g_{n})_{n\ge 0}$ as functions on ${\mathbb {R}}_{+}$ via $t\longmapsto g_{[t]}$, and write $g(t)\sim h(t)$ as $t\rightarrow \infty$ if $g(t)/h(t)\rightarrow 1$. Moreover, $g(t)\asymp h(t)$ means $0< {{\underline{\lim }}}_{t\rightarrow \infty }\, g(t)/h(t) \le {{\overline{\lim }}}_{t\rightarrow \infty }\, g(t)/h(t) < \infty$.

### Theorem 2.3

(Relative size of digits and partial sums)  

**a)** We have

2.9 $$\begin{aligned} \underset{n\rightarrow \infty }{{\overline{\lim }}}\,\frac{{\textsf{a}}_{n}^{\prime } }{\sum _{k=1}^{n-1}{\textsf{a}}_{k}^{\prime }}=\,\infty \text { a.e.} \end{aligned}$$

Generally, for functions $g:[0,\infty )\rightarrow (3,\infty )$ fulfilling $g(\eta (t))\asymp g(t)$ if $\eta (t) \sim t$ as $t\rightarrow \infty$, we have

2.10 $$\begin{aligned} \underset{n\rightarrow \infty }{{\overline{\lim }}}\,\frac{g({\textsf{a}}_{n} ^{\prime })}{\sum _{k=1}^{n-1}{\textsf{a}}_{k}^{\prime }}=\infty \text { a.e. iff }\int _{c}^{\infty }\frac{g(y)}{\log _{2}g(y)}\frac{dy}{y^{2}\log y}=\infty \text {,} \end{aligned}$$

while otherwise

$$\begin{aligned} \lim _{n\rightarrow \infty }\,\frac{g({\textsf{a}}_{n}^{\prime })}{\sum _{k=1} ^{n-1}{\textsf{a}}_{k}^{\prime }}=0\text { a.e.} \end{aligned}$$

**b)** In contrast, comparing to the unrestricted digit sum $\sum _{k=1}^{n-1}{\textsf{a}}_{k}$, one has

$$\begin{aligned} \lim _{n\rightarrow \infty }\,\frac{{\textsf{a}}_{n}^{\prime }}{\sum _{k=1} ^{n-1}{\textsf{a}}_{k}}=0\text { a.e.} \end{aligned}$$

Generally, for functions $g:[0,\infty )\rightarrow (3,\infty )$ fulfilling $g(\eta (t))\asymp g(t)$ if $\eta (t) \sim t$ as $t\rightarrow \infty$, we have

$$\begin{aligned} \underset{n\rightarrow \infty }{{\overline{\lim }}}\,\frac{g({\textsf{a}}_{n} ^{\prime })}{\sum _{k=1}^{n-1}{\textsf{a}}_{k}}=\infty \text { a.e. iff }\int _{c}^{\infty }\frac{g(y)}{\log g(y)}\frac{dy}{y^2\log y} =\infty \text {,} \end{aligned}$$

while otherwise

$$\begin{aligned} \lim _{n\rightarrow \infty }\,\frac{g({\textsf{a}}_{n}^{\prime })}{\sum _{k=0} ^{n-1}{\textsf{a}}_{k}}=0\text { a.e.} \end{aligned}$$

**c)** Turning to a comparison of partial sums, we find that

$$\begin{aligned} \lim _{n\rightarrow \infty }\,\frac{\sum _{k=1}^{n}{\textsf{a}}_{k}^{\prime }}{\sum _{k=1}^{n}{\textsf{a}}_{k}}=0\text { a.e.} \end{aligned}$$

### Remark 2.2

A broad class of functions which satisfy $g(\eta (t))\asymp g(t)$ if $\eta (t) \sim t$ as $t\rightarrow \infty$, are the regularly varying functions. Recall that a measurable function $g:(L,\infty )\rightarrow (0,\infty )$ is *regularly varying of index* $\rho \in {\mathbb {R}}$ at infinity, written $g\in {\mathcal {R}}_{\rho }$, if $g(ct)/g(t)\rightarrow c^{\rho }$ as $t\rightarrow \infty$ for all $c>0$ (see Chapter 1 of [6] for more information).

Whether or not the integrals diverge can easily be checked for many specific *g*’s:

### Example 2.2

**a)** Taking $g(t):=t\,\log ^{\rho }t$, $\rho \in {\mathbb {R}}$, part b) gives

$$\begin{aligned} \underset{n\rightarrow \infty }{{\overline{\lim }}}\,\frac{{\textsf{a}}_{n}^{\prime }\log {\textsf{a}}_{n}^{\prime }}{\sum _{k=1}^{n-1}{\textsf{a}}_{k}}=\infty \text { a.e. for }\rho >1\quad \text { while }\quad \lim _{n\rightarrow \infty }\,\frac{{\textsf{a}} _{n}^{\prime }\log ^{\rho }{\textsf{a}}_{n}^{\prime }}{\sum _{k=1}^{n-1} {\textsf{a}}_{k}}=0\text { a.e. for }\rho \le 1\text {.} \end{aligned}$$

**b)** In case $g(t):=t\,\log t/\log _{2}^{\gamma }t$, $\gamma \in {\mathbb {R}}$, we find for $\gamma \le 1$

$$\begin{aligned} \underset{n\rightarrow \infty }{{\overline{\lim }}}\,\frac{{\textsf{a}}_{n}^{\prime }\log {\textsf{a}}_{n}^{\prime }/\log _{2}{\textsf{a}}_{n}^{\prime }}{\sum _{k=1} ^{n-1}{\textsf{a}}_{k}}=\infty \text { a.e.} \end{aligned}$$

while, for $$\gamma >1$$,

$$\begin{aligned} \lim _{n\rightarrow \infty }\,\frac{{\textsf{a}}_{n}^{\prime }\log {\textsf{a}} _{n}^{\prime }/\log _{2}^{\gamma }{\textsf{a}}_{n}^{\prime }}{\sum _{k=1} ^{n-1}{\textsf{a}}_{k}}=0\text { a.e.} \end{aligned}$$

On the other hand, if we look at primes to some power $\gamma$ we obtain—as a counterpart to Theorem 2.2 b)—the following result:

### Theorem 2.4

**a)** For $\gamma <1$ there exists $K_\gamma >0$ such that

$$\begin{aligned} \lim _{n\rightarrow \infty }\,\frac{\sum _{k=1}^{n}\left( {\textsf{a}}_{k}^{\prime }\right) ^{\gamma }}{n}=K_\gamma <\infty \text { a.e.} \end{aligned}$$

**b)** Let $\sigma :=\sigma _{(n,x)}\in {\mathcal {S}}_n$ be a pointwise permutation, i.e. $\sigma : I\times \{1,\ldots , n\} \rightarrow \{1,\ldots , n\}$, such that ${\textsf{a}}_{\sigma (1)}^{\prime }\ge \ldots \ge {\textsf{a}}_{\sigma (n)}^{\prime }$ and ${\textsf{S}}_n^k:=\sum _{j=k+1}^{n}{\textsf{a}}_{\sigma (j)}^{\prime }$. If $\gamma >1$, then for all $(b_n)\in {\mathbb {N}}^{{\mathbb {N}}}$ fulfilling $b_n=o(n^{1-\epsilon })$ for some $\epsilon >0$ and

2.11 $$\begin{aligned} \lim _{n\rightarrow \infty }\frac{b_n}{\log \log n}=\infty \end{aligned}$$

we have

2.12 $$\begin{aligned} \lim _{n\rightarrow \infty }\,\frac{{\textsf{S}}_n^{b_n}}{d_n}= 1\text { a.e.} \end{aligned}$$

where

2.13 $$\begin{aligned} d_n\sim \frac{1}{(\gamma -1)(\log 2)^{\gamma }}\cdot \frac{n^{\gamma }b_n^{1-\gamma }}{(\log n)^{\gamma }}. \end{aligned}$$

### Remark 2.3

It is not proven that a trimming rate slower than the one given in (2.11) is possible. However, by [11] one can deduce that for i.i.d. random variables with the same distribution function and $b_n\asymp \log \log n$ a strong law of large numbers as in (2.12) is no longer possible.

However, if we only ask for convergence in probability, the picture looks much simpler and we refer the reader to Theorem 3.1 in the next section.

## Main results—distributional matters

The second set of results we present focuses on the distributions of (various functions of) the digits ${\textsf{a}}_{n}^{\prime }$. If (*M*, *d*) is a separable metric space with Borel $\sigma$-field ${\mathcal {B}}_{M}$, a sequence $(\nu _{n})_{n\ge 1}$ of probability measures on $(M,{\mathcal {B}}_{M})$ *converges weakly* to the probability measure $\nu$ on $(M,{\mathcal {B}} _{M})$, written $\nu _{n}\Longrightarrow \nu$, if the integrals of bounded continuous function $\psi :M\rightarrow {\mathbb {R}}$ converge, i.e. $\int \psi \,d\nu _{n}\longrightarrow \int \psi \,d\nu$ as $n\rightarrow \infty$. If $R_{n}:I\rightarrow M$, $n\ge 1$, Borel measurable functions and $\nu$ a Borel probability on *M* (or *R* another *random element* of *M*, not necessarily defined on *I*, with distribution $\nu$) then $(R_{n} )_{n\ge 1}$ *converges in distribution* to $\nu$ (or to *R*) *under the probability measure* *P* on ${\mathcal {B}}_{I}$, if the distributions $P\circ R_{n}^{-1}$ of the $R_{n}$ w.r.t. *P* converge weakly to $\nu$. Explicitly specifying the underlying measure, we denote this by

$$\begin{aligned} R_{n}\overset{P}{\Longrightarrow }\nu \text { or }R_{n}\overset{P}{\Longrightarrow }R\text {.} \end{aligned}$$

For sequences $(R_{n})$ defined on an ergodic dynamical system, it is often the case that a distributional limit theorem $R_{n}\overset{P}{\Longrightarrow }R$ automatically carries over to a large collection of other probability measures: *strong distributional convergence*, written

$$\begin{aligned} R_{n}\overset{{\mathcal {L}}(\lambda )}{\Longrightarrow }\nu \text { or }R_{n}\overset{{\mathcal {L}}(\lambda )}{\Longrightarrow }R\text {,} \end{aligned}$$

means that $R_{n}\overset{P}{\Longrightarrow }R$ for all probability measures $P\ll \lambda$, see [24].

We start by giving a counterpart to Theorem 2.4 for weak convergence, where b) is in the spirit of [19].

### Theorem 3.1

**a)** For $\gamma <1$ there exists $K_\gamma >0$ such that

$$\begin{aligned} \frac{\sum _{k=1}^{n}\left( {\textsf{a}}_{k}^{\prime }\right) ^{\gamma }}{n}\overset{{\mathcal {L}}(\lambda )}{\Longrightarrow } K_\gamma . \end{aligned}$$

**b)** For the case $\gamma =1$ we have

$$\begin{aligned} \frac{\sum _{k=1}^{n}{\textsf{a}}_{k}^{\prime }}{n\log _2 n}\overset{{\mathcal {L}}(\lambda )}{\Longrightarrow } \log 2. \end{aligned}$$

**c)** If $\gamma >1$ we have

$$\begin{aligned} \frac{{\textsf{S}}_n^{b_n}}{d_n}\overset{{\mathcal {L}}(\lambda )}{\Longrightarrow } 1, \end{aligned}$$

where ${\textsf{S}}_n^{b_n}$ is defined as in Theorem 2.4, $(d_n)$ is given as in (2.13) and $\lim _{n\rightarrow \infty }b_n=\infty$ and $b_n=o(n^{1-\epsilon })$.

### Remark 3.1

Indeed by [17] the stronger result of convergence in mean follows for c). It is not proven that for the situation in c) convergence in probability can not hold for a lightly trimmed sum, i.e. a sum from which only a finite number of large entries, being independent of *n* is removed. However, it follows from [1] that $\sum _{k=1}^n({\textsf{a}}_k^{\prime })^{\gamma }$ normed by the right norming sequence converges to a non-degenerate Mittag-Leffler distribution if $\gamma >1$. On the other hand, by [18] it follows that light trimming does not have any influence on distributional convergence if the random variables considered are i.i.d.

As we have seen in the previous section, the maximum ${\textsf{M}}_{n}^{\prime }$ has a large influence then the whole system, in the following we will give its distributional convergence. We let $\Theta$ denote a positive random variable with $\Pr [\Theta \le y]=e^{-1/y}$, $y>0$ and get the following counterpart to [21].

### Theorem 3.2

(Distributional convergence of ${\textsf{M}}_{n}^{\prime }$ )The maximum ${\textsf{M}}_{n}^{\prime }$ of the prime digits converges in distribution,

3.1 $$\begin{aligned} \frac{\log 2\,\log n}{n}\cdot {\textsf{M}}_{n}^{\prime }\overset{{\mathcal {L}} (\lambda )}{\Longrightarrow }\Theta \text { }\quad \text {as }n\rightarrow \infty \text {.} \end{aligned}$$

A related classical topic, introduced by Doeblin [9], is the Poissonian nature of occurrences of very large CF-digits. For $l\ge 1$ let $\varphi _{l}=\varphi _{l,1}:=\inf \{k\ge 1:{\textsf{a}}_{k}\ge l\}$, the first position in the CF-expansion at which a digit $\ge l$ shows up, and $\varphi _{l,i+1}:=\inf \{k\ge 1:{\textsf{a}}_{\varphi _{l,i}+k}\ge l\}$ the distance between the *i*th and $(i+1)$st occurrence. Defining $\Phi _{l}:I\rightarrow [0,\infty ]^{{\mathbb {N}}}$ as $\Phi _{l}:=(\varphi _{l,1},\varphi _{l,2},\ldots )$ and letting $\Phi _{\textrm{Exp}}$ denote an i.i.d. sequence of normalized exponentially distributed random variables, we can express this classical result by stating that

$$\begin{aligned} \frac{1}{\log 2}\frac{1}{l}\cdot \Phi _{l}\overset{\lambda }{\Longrightarrow }\,\Phi _{\textrm{Exp}}\quad \text {as }l\rightarrow \infty . \end{aligned}$$

Turning to prime digits, we shall consider the corresponding quantities $\varphi _{l,i}^{\prime }$ with $\varphi _{l,0}^{\prime }:=0$ and $\varphi _{l,i+1}^{\prime }:=\inf \{k\ge 1:{\textsf{a}}_{\varphi _{l,i}^{\prime }+k}^{\prime }\ge l\}$, $i\ge 0$, and the processes $\Phi _{l}^{\prime }:=(\varphi _{l,1}^{\prime },\varphi _{l,2}^{\prime },\ldots )$ of distances between consecutive occurrences of prime digits of size at least *l*. In fact, we also provide refined versions of the limit theorem which show that, asymptotically, both the relative size compared to *l* of such a large prime digit ${\textsf{a}}_{\varphi _{l,i}^{\prime }}^{\prime }$ and its residue class for a given modulus *m*, are stochastically independent of the positions $\varphi _{l,i}^{\prime }$ at which they occur. (These statements are parallel to Propositions 10.1 and 10.2 of [25]. A $(q_1,\ldots ,q_d)$-Bernoulli sequence is an iid sequence of random variables which can assume *d* different values with respective probabilities $q_1,\ldots ,q_d$.)

### Theorem 3.3

(Poisson limits for large prime CF-digits) The sequences $\Phi _{l}^{\prime }$ of positions at which large prime digits occur satisfy the following.

**a)** Their distances converge to an i.i.d. sequence of exponential variables,

3.2 $$\begin{aligned} \frac{1}{\log 2}\frac{1}{l\log l}\cdot \Phi _{l}^{\prime }\overset{{\mathcal {L}}(\lambda )}{\Longrightarrow }\,\Phi _{\textrm{Exp}}\quad \text {as } l\rightarrow \infty . \end{aligned}$$

**b)** Take any $\vartheta \in (0,1)$, let $$\psi _{l,i}^{\prime }\ $$be the indicator function of $\{{\textsf{a}}_{\varphi _{l,i}^{\prime }}^{\prime }\ge l/\vartheta \}$ and set $\Psi _{l}^{\prime }:=(\psi _{l,1}^{\prime },\psi _{l,2}^{\prime },\ldots )$, which identifies those prime digits $\ge l$ which are in fact $\ge l/\vartheta$. Then

3.3 $$\begin{aligned} \left( \frac{1}{\log 2}\frac{1}{l\log l}\cdot \Phi _{l}^{\prime },\Psi _{l}^{\prime }\right) \overset{{\mathcal {L}}(\lambda )}{\Longrightarrow } (\,\Phi _{\textrm{Exp}},\Psi ^{\prime })\quad \text {as }l\rightarrow \infty \text {,} \end{aligned}$$

where $(\,\Phi _{\textrm{Exp}},\Psi ^{\prime })$ is an independent pair with $\Psi ^{\prime }$ a $({ 1-\vartheta },\vartheta )$-Bernoulli sequence.

**c)** Fix an integer $m\ge 2$. For $l>m$ define $\upsilon _{l,i}^{\prime }:I\rightarrow \{j\in \{1,\ldots ,m\}:j$ relatively prime to $m\}$ by $\upsilon _{l,i}^{\prime }(x):=j$ if ${\textsf{a}}_{\varphi _{l,i}^{\prime }}^{\prime }(x)\equiv j \mod m$, so that $\Upsilon _{l}^{\prime }:=(\upsilon _{l,1}^{\prime },\upsilon _{l,2}^{\prime },\ldots )$ identifies the residue classes mod *m* of the prime digits ${\textsf{a}} _{\varphi _{l,i}^{\prime }}^{\prime }$. Then

3.4 $$\begin{aligned} \left( \frac{1}{\log 2}\frac{1}{l\log l}\cdot \Phi _{l}^{\prime },\Upsilon _{l}^{\prime }\right) \overset{{\mathcal {L}}(\lambda )}{\Longrightarrow } (\,\Phi _{\textrm{Exp}},\Upsilon ^{\prime })\quad \text {as }l\rightarrow \infty \text {,} \end{aligned}$$

where $(\,\Phi _{\textrm{Exp}},\Upsilon ^{\prime })$ is an independent pair with $\Upsilon ^{\prime }$ a $\big (\frac{1}{\phi (m)},\ldots ,\frac{1}{\phi (m)} \big )$-Bernoulli sequence. (Here $\phi (m)$ denotes the Euler totient.)

We finally look at the distribution of a function which counts how many ${\textsf{a}}_n^{\prime }$ fall into particular sets $A_n$ giving a limit theorem in the spirit of [16, 20]. We let ${\mathcal {N}}$ denote a positive random variable with $\Pr [{\mathcal {N}}\le y]=\int _0^y e^{-t^2/2}\,\textrm{d}t/\sqrt{2\pi }$, $y>0$.

### Theorem 3.4

(A CLT for counting primes in CF) Suppose that either

(A) $$A_n{:}{=}\left\{ {\textsf{a}}_n^{\prime }\ge b_n\right\} $$ with $$(b_n)\in {\mathbb {R}}^{{\mathbb {N}}}$$ and $$\sum _{n:b_{n}>1} {1}/{b_n\log b_n} =\infty $$,
(B) $A_n{:}{=}{\left\{ {\textsf{a}}_n^{\prime }= d_n\right\} }$ with $(d_n)$ a sequence of primes and $\sum _{n\in {\mathbb {N}}}{1}/{d_{n}^2}=\infty$,
(C) $A_n{:}{=}\left\{ d_n\le {\textsf{a}}_n^{\prime } \le d_n \cdot \left( 1+\frac{1}{c_n}\right) \right\}$ with $(d_n)$ a sequence of natural numbers tending to infinity, $(c_n)$ a sequence of positive numbers with $c_n \le d_n^{0.475}$ and $\sum _{n=1}^{\infty }{1}/{\left( c_n d_n\log (d_n)\right) }=\infty$.

Then, for $S_n{:}{=}\sum _{k=1}^n\mathbbm {1}_{A_k}$ the following central limit theorem holds:

$$\begin{aligned} \frac{S_n-\int S_n\,\textrm{d}\mu _{{\mathfrak {G}}}}{ \sqrt{ \int \left( S_n-\int S_n\,\textrm{d}\mu _{{\mathfrak {G}}}\right) ^2}}\overset{{\mathcal {L}} (\lambda )}{\Longrightarrow }{\mathcal {N}}\text { }\quad \text {as }n\rightarrow \infty . \end{aligned}$$

## The Gauss map and the prime digit function

The results announced above express properties of certain stochastic processes derived from the exceptionally well understood dynamical system generated by the ergodic *continued fraction map* (or *Gauss map*)

$$\begin{aligned} S:(0,1]\rightarrow [0,1]\text {, }Sx:=\frac{1}{x}-\left\lfloor \frac{1}{x}\right\rfloor =\frac{1}{x}-k\text { for }x\in \left( \frac{1}{k+1},\frac{1}{k}\right] =:I_{k}\text {, }k\ge 1 \end{aligned}$$

which, since [10], is known to preserve the probability density

$$\begin{aligned} h_{{\mathfrak {G}}}(x):=\frac{1}{\log 2}\frac{1}{1+x}\text {, }x\in I\text {.} \end{aligned}$$

The invariant *Gauss measure* $\mu _{{\mathfrak {G}}}$ on ${\mathcal {B}}_{I}$ defined by the latter, $\mu _{{\mathfrak {G}}}(B):=\int _{B}h_{{\mathfrak {G}} }(x)\,dx$, is exact (and hence ergodic). As hardly any textbook on ergodic theory fails to point out, iteration of *S* reveals the continued fraction digits of any $x\in I$, in that

$$\begin{aligned} x=\left[ {\textsf{a}}_{1}(x),{\textsf{a}}_{2}(x),\ldots \right] \text { with }{\textsf{a}}_{n}(x)={\textsf{a}}\circ S^{n-1}(x)\text {, }n\ge 1\text {,} \end{aligned}$$

where ${\textsf{a}}:I\rightarrow {\mathbb {N}}$ is the *digit function* corresponding to the partition $\xi :=\{I_{k}:k\ge 1\}$, i.e. ${\textsf{a}} (x):=\left\lfloor 1/x\right\rfloor =k$ for $x\in I_{k}$. The stationary sequence $({\textsf{a}}\circ S^{n})_{n\ge 0}$ on the probability space $(I,{\mathcal {B}}_{I},\mu _{{\mathfrak {G}}})$ thus obtained exhibits interesting properties since ${\textsf{a}}$ has infinite expectation, $\int _{I} {\textsf{a}}\,d\mu _{{\mathfrak {G}}}=\sum _{k\ge 1}k\,\mu _{{\mathfrak {G}}} (I_{k})=\infty$, as $\mu _{{\mathfrak {G}}}(I_{k})=\log (\frac{(k+1)^{2}}{k(k+2)})/\log 2\sim 1/(\log 2\cdot k^{2})$ for $k\rightarrow \infty$. As in classical probability theory, the *tail behaviour of the distribution*, given by

$$\begin{aligned} \mu _{{\mathfrak {G}}}\left( \left\{ {\textsf{a}}\ge K\right\} \right) =\frac{1}{\log 2}\cdot \log \left( \frac{K+1}{K}\right) \sim \frac{1}{\log 2}\cdot \frac{1}{K}\text { as }K\rightarrow \infty \end{aligned}$$

(which entails $L(N):=\int _{I}({\textsf{a}}\wedge N)\,d\mu _{{\mathfrak {G}}} =\sum _{K=1}^{N}\mu _{{\mathfrak {G}}}\left( \left\{ {\textsf{a}}\ge K\right\} \right) \sim \log N/\log 2$ as $N\rightarrow \infty$), is the key to fine asymptotic results. However, the study of the CF digit sequence goes beyond standard results, since the random variables ${\textsf{a}}\circ S^{n}$ are not independent. Yet, it is well known that they still satisfy a strong form of *asymptotic independence* or *mixing* in the following sense:

Given any measure preserving transformation *T* on a probability space $(\Omega ,{\mathcal {B}},P)$, and a countable measurable partition $\gamma$ (mod *P*), the $\psi$*-mixing coefficients of* $\gamma$ are defined as

$$\begin{aligned} \psi _{\gamma }(n):=\sup _{k\ge 1}\left\{ \left| \frac{P(V\cap W)}{P(V)P(W)}-1\right| : \begin{array}{ll} V\in \sigma (\bigvee _{j=0}^{k-1}T^{-j}\gamma ),P(V)>0, &{} \\ W\in T^{-(n+k-1)}{\mathcal {B}},P(W)>0 &{} \end{array} \right\} \text {, }n\ge 1\text {.} \end{aligned}$$

The partition $\gamma$ is said to be *continued-fraction (CF-) mixing* for the probability preserving system $(\Omega ,{\mathcal {B}},P,T)$ if it is generating, and if $\psi _{\gamma }(1)<\infty$ as well as $\psi _{\gamma }(n)\rightarrow 0$ for $n\rightarrow \infty$. (Note that $(\psi _{\gamma }(n))_{n\ge 1}$ is non-increasing.) Of course, the nomenclature is due to the fact that

4.1 $$\begin{aligned} \xi \text { is CF-mixing for }(I,{\mathcal {B}}_{I},\mu _{{\mathfrak {G}}},S)\text {.} \end{aligned}$$

Actually, this system is *exponentially CF-mixing*, in that there are constants $C>0$ and $\rho \in (0,1)$ such that

$$\begin{aligned} \psi _{\xi }(n)\le C\,\rho ^{n}\text { for }n\ge 1 \end{aligned}$$

(which is related to Gauss’ famous question mentioned in the introduction, see e.g. [13] or [23]).

We are going to study occurrences of *prime digits* by considering the *restricted digit function* ${\textsf{a}}^{\prime }:= (\mathbbm {1}_{{\mathbb {P}}} \circ {\textsf{a}}) \cdot {\textsf{a}}:I\rightarrow \{0\}\cup {\mathbb {P}}$. As in the case of ${\textsf{a}}$, this function, as a random variable on $(I,{\mathcal {B}}_{I},\mu _{{\mathfrak {G}}})$, still has infinite expectation. Indeed, the prime number theorem (PNT) enables us to quickly determine the all-important tail asymptotics for the distribution of ${\textsf{a}}^{\prime }$. The following lemma is the key to our analysis of the prime digit sequence.

### Lemma 4.1

(Tail behaviour and truncated expectation of ${\textsf{a}}^{\prime }$)The distribution of ${\textsf{a}}^{\prime }$ (with respect to the Gauss measure) satisfies

4.2 $$\begin{aligned} \mu _{{\mathfrak {G}}}\left( \left\{ {\textsf{a}}^{\prime }\ge K\right\} \right) \sim \frac{1}{\log 2}\cdot \frac{1}{K\log K}\text { as }K\rightarrow \infty \text {.} \end{aligned}$$

In particular, ${\textsf{a}}^{\prime }$ is not integrable, $\int _{I} {\textsf{a}}^{\prime }\,d\mu _{{\mathfrak {G}}}=\infty$. Moreover,

4.3 $$\begin{aligned} L^{\prime }(N):=\int _{I}({\textsf{a}}^{\prime }\wedge N)\,d\mu _{{\mathfrak {G}}} \sim \frac{\log _{2}N}{\log 2}\text { as }N\rightarrow \infty \text {.} \end{aligned}$$

so that $a^{\prime }(N):=N/L^{\prime }(N)\sim \log 2\cdot N/\log _{2}N$ is asymptotically inverse to $b^{\prime }(N):=(N\,\log _{2}N)/\log 2$.

### Proof

First, the PNT is easily seen (cf. [12], Theorem 1.8.8) to imply that

4.4 $$\begin{aligned} \textrm{p}_{n}\sim n\log n\text { as }n\rightarrow \infty \text {,} \end{aligned}$$

where $\textrm{p}_{n}$ denotes the *n*th prime number. Therefore,

$$\begin{aligned} \sum _{n\ge N}\frac{1}{\textrm{p}_{n}^{2}}\sim \sum _{n\ge N}\frac{1}{n^{2}(\log n)^{2}}\sim \frac{1}{N(\log N)^{2}}\text { as }N\rightarrow \infty \text {.} \end{aligned}$$

Letting *N*(*K*) denote the least *n* with $\textrm{p}_{n}\ge K$, we have, as $K\rightarrow \infty$,

$$\begin{aligned} \mu _{{\mathfrak {G}}}\left( \left\{ {\textsf{a}}^{\prime }\ge K\right\} \right) =\sum _{\textrm{p}\ge K,\textrm{p}\in {\mathbb {P}}}\mu _{{\mathfrak {G}} }(I_{\textrm{p}})\sim \frac{1}{\log 2}\sum _{\textrm{p}\ge K,\textrm{p} \in {\mathbb {P}}}\frac{1}{\textrm{p}^{2}}=\frac{1}{\log 2}\sum _{n\ge N(K)} \frac{1}{\textrm{p}_{n}^{2}} \end{aligned}$$

and, by PNT, $N(K)\sim K/\log K$. Combining these observations yields (4.2). The second statement is an easy consequence thereof, since

$$\begin{aligned} L^{\prime }(N)=\sum _{K=1}^{N}\mu _{{\mathfrak {G}}}\left( \left\{ {\textsf{a}} ^{\prime }\ge K\right\} \right) \sim \frac{1}{\log 2}\sum _{K=2}^{N}\frac{1}{K\log K}\sim \frac{1}{\log 2}\int _{2}^{N}\frac{dx}{x\log x}\text { as }N\rightarrow \infty \text {.} \end{aligned}$$

Straightforward calculation verifies the assertions about $a^{\prime }$ and $b^{\prime }$. $\square$

### Remark 4.1

Several of the results allow for analogues in which prime digits are replaced by digits belonging to other subsets ${\mathbb {M}}$ of the integers for which $\pi _{{\mathbb {M}}}(n):=\#{\mathbb {M}}\cap \{1,\ldots ,n\}$ is regularly varying with $\sum _{m\in {\mathbb {M}}}\frac{1}{m}=\infty$, like, for example, the set of integers which are the product of exactly *k* prime numbers, see Theorem 3.5.11 of [14]. (M. Thaler, personal communication.)

## Proofs of the results on a.e. convergence

We are now ready for the proofs of our pointwise convergence results. We can always work, without further mention, with the invariant measure $\mu _{{\mathfrak {G}}}$, since it has the same null-sets as $\lambda$.

### Proof of Proposition 2.1

This, of course, is just the ergodic theorem,

$$\begin{aligned} \frac{1}{n}\sum _{k=1}^{n}\mathbbm {1}_{{\mathbb {P}}}\circ {\textsf{a}}_{k}=\frac{1}{n} \sum _{k=0}^{n-1}\mathbbm {1}_{{\mathbb {P}}}\circ S^{k}\longrightarrow \mu _{{\mathfrak {G}}} ({\mathbb {P}})=\sum _{\textrm{p}\in {\mathbb {P}}}\mu _{{\mathfrak {G}}}(I_{\textrm{p}})\text { a.e. as }n\rightarrow \infty \text {.} \end{aligned}$$

$$\square $$

In the following we will repeatedly appeal to the following version of Rényi’s Borel-Cantelli Lemma (BCL) (as in Lemma 1 of [3]):

### Lemma 5.1

(Rényi’s Borel-Cantelli Lemma)Assume that $(E_{n})_{n\ge 1}$ is a sequence of events in the probability space $(\Omega ,{\mathcal {B}},P)$ for which there is some $r\in (0,\infty )$ such that

$$\begin{aligned} \frac{P(E_{j}\cap E_{k})}{P(E_{j})\,P(E_{k})}\le r\qquad \text {whenever }j,k\ge 1\text {, }j\ne k\text {.} \end{aligned}$$

Then $P(\{E_{n}$ infinitely often$\})>0$ iff $\sum _{n\ge 1}P(E_{n})=\infty$.

This lemma enables us to prove Theorem 2.1.

### Proof of Theorem 2.1

**a)** Note that $\{{\textsf{a}}_{j}^{\prime }>c\}=S^{-(j-1)}\{{\textsf{a}}^{\prime }>c\}$ with $\{{\textsf{a}}^{\prime }>c\}$ measurable w.r.t. $\xi$. As a consequence of the CF-mixing property (4.1), we see that Rényi’s BCL applies to show that

5.1 $$\begin{aligned} \mu _{{\mathfrak {G}}}(\{{\textsf{a}}_{n}^{\prime }>b_{n}\text { i.o.}\})>0\text { iff }\sum _{n\ge 1}\mu _{{\mathfrak {G}}}(\{{\textsf{a}}_{n}^{\prime }>b_{n}\})=\infty \text {.} \end{aligned}$$

By *S*-invariance of $\mu _{{\mathfrak {G}}}$ and Lemma 4.1, we have $\mu _{{\mathfrak {G}}}(\{{\textsf{a}}_{n}^{\prime }\ge b_{n}\})=\mu _{{\mathfrak {G}}}(\{{\textsf{a}}^{\prime }\ge b_{n}\})\sim 1/(b_{n}\log b_{n})$, so that divergence of the right-hand series in (5.1) is equivalent to that of $\sum _{n\ge 1}(b_{n}\log b_{n})^{-1}$. Finally, again because of $\{{\textsf{a}}_{j}^{\prime }>c\}=S^{-(j-1)}\{{\textsf{a}}^{\prime }>c\}$, the set$\ \{{\textsf{a}}_{n}^{\prime }>b_{n}$ i.o.$\}$ is easily seen to belong to the tail-$\sigma$-field $\bigcap _{n\ge 0}S^{-n}{\mathcal {B}}_{I}$ of *S*. The system $(I,{\mathcal {B}}_{I},\mu _{{\mathfrak {G}}},S)$ being exact, the latter is trivial mod $\mu _{{\mathfrak {G}}}$. Hence $\mu _{{\mathfrak {G}}}(\{{\textsf{a}} _{n}^{\prime }>b_{n}$ i.o.$\})>0$ implies $\mu _{{\mathfrak {G}}}(\{{\textsf{a}} _{n}^{\prime }>b_{n}$ i.o.$\})=1$.

**b) **Statement (2.2) is seen by an easy routine argument, as in the proof of Proposition 3.1.8 of [13].

**c) **Without loss of generality we first assume that $c_n\le 0.5$, for all *n*. If this doesn’t hold, we can easily switch to a subsequence in which this holds and consider the subsequences separately. By the prime number theorem we have

$$\begin{aligned} \lambda ({\textsf{a}}_{1}^{\prime }\in [d_n, d_n(1+1/c_n)])&= \lambda ({\textsf{a}}_{1}^{\prime }\ge d_n) -\lambda ({\textsf{a}}_{1}^{\prime }\ge d_n(1+1/c_n))\\&\asymp \frac{1}{d_n\log d_n}-\frac{1}{d_n(1+1/c_n)\log (d_n(1+1/c_n))}\\&\sim \frac{1}{c_nd_n\log d_n}. \end{aligned}$$

Next, we assume that $c_n>0.5$. We note that

5.2 $$\begin{aligned} { \# }{\mathbb {P}}\cap (d_n, d_n(1+1/c_n)]\cdot \lambda ({\textsf{a}}_{1}=d_n)&\le \lambda ({\textsf{a}}_{1}^{\prime }\in [d_n, d_n(1+1/c_n)])\nonumber \\&\le \#{\mathbb {P}}\cap (d_n, d_n(1+1/c_n)]\cdot \lambda ({\textsf{a}}_{1}=d_n(1+1/c_n)). \end{aligned}$$

Furthermore,

5.3 $$\begin{aligned} \lambda ({\textsf{a}}_{1}=d_n)\asymp \frac{1}{d_n^2}\asymp \lambda ({\textsf{a}}_{1}=d_n(1+1/c_n)). \end{aligned}$$

On the other hand, we have by [4], p. 562 that there exists $K>0$ such that

$$\begin{aligned} \#{\mathbb {P}}\cap (d_n, d_n(1+1/c_n)]&\sim \pi (d_n(1+1/c_n))-\pi (d_n) \le K\cdot \frac{d_n}{c_n\log d_n}. \end{aligned}$$

Combining this with (5.2) and () yields the statement of c).

**d) ** This follows immediately from [16, Theorem 6a]. $\square$

### Proof of Lemma 2.1

By assumption there is some $\varepsilon \in (0,1)$ such that the set $M:= \{ n \ge 1: (n \log _2 n)/b_n \ge \varepsilon \}$ is infinite. Define $c(x):=\exp (\sqrt{\log x})$ and $f(x):=x \log x \log _2 x$ for $x>1$, and note that $c(x)<x$ for $x>e$. Suppose that $n\in M$, $n \ge 4$, and $c(n)<k\le n$. Since $k\le n$, we have $b_k = (b_k/k)k \le (b_n/n)k \le (1/\varepsilon ) k \log _2 n$, and thus

$$\begin{aligned} b_k \log b_k&\le (1/\varepsilon ) k \log _2 n (\log (1/\varepsilon ) +\log k + \log _3 n)\\&= \frac{f(k)}{\varepsilon } \frac{\log _2 n}{\log _2 k} \left( - \frac{\log (\varepsilon )}{ \log k} + 1 + \frac{\log _3 n}{\log k} \right) . \end{aligned}$$

On the other hand, $c(n)<k$ implies $\log k > \sqrt{\log n}$ and hence

$$\begin{aligned} \log _2 k> (1/2) \log _2 n,\; \log k> 1, \;\text { and }\; \log k > \log _3 n. \end{aligned}$$

Using these estimates we see that

$$\begin{aligned} b_k \log b_k \le \frac{f(k)}{C(\varepsilon )} \;\;\;\text { with } \;\;\; C(\varepsilon ):= \frac{\varepsilon }{2 \left( -\log (\varepsilon )+2 \right) } >0. \end{aligned}$$

Taking into account that $\log _3 x$ is a primitive of 1/*f*(*x*) we get

$$\begin{aligned} \sum _{k>c(n)} \frac{1}{b_k \log b_k}&\ge C(\varepsilon ) \sum _{c(n)<k\le n} \frac{1}{f(k)}\\&\ge C(\varepsilon ) \left( \int _{c(n)}^{n} \frac{dx}{f(x)} - \frac{1}{f( \left[ c(n) \right] )} \right) = C(\varepsilon ) \left( \log 2 - \frac{1}{f( \left[ c(n) \right] )} \right) . \end{aligned}$$

Since this estimate holds for infinitely many *n*, we see that

$$\begin{aligned} \underset{n\rightarrow \infty }{{\overline{\lim }}}\, \sum _{k>n} \frac{1}{b_k \log b_k} \ge C(\varepsilon ) \log 2, \end{aligned}$$

proving that $\sum _{k \ge 1} \frac{1}{b_k \log b_k}$ diverges.

### Proof of Theorem 2.2

**a)** Since $\int _{I} {\textsf{a}}^{\prime }\,d\mu _{{\mathfrak {G}}}=\infty$ by Lemma 4.1, this is immediate from the ergodic theorem. **b) **We apply Theorem 1.1 of [2] to $(I,{\mathcal {B}}_{I},\mu _{{\mathfrak {G}}},S)$ and ${\textsf{a}}^{\prime }$, observing that (in the notation of that paper), ${\mathfrak {N}}_{{\textsf{a}}^{\prime }}=1$ since $J_{1}=\sum _{n\ge 1}\left( n^{2}\,\log n\,\log _{2}n\right) ^{-1}<\infty$. Furthermore, by using the estimate of Lemma 4.1 and setting $a^{\prime }(N):=N/L^{\prime }(N)\sim \log 2\cdot N/\log _{2}N$ we get that its asymptotic inverse can be written as $b^{\prime }(N):=(N\,\log _{2}N)/\log 2$ which by the statement of the paper coincides with the norming sequence.

**c) ** Using Theorem 2.1 a), we first note that $\sum _{n\ge 1}1/(b_{n}\log b_{n})=\infty$ implies $\overline{\lim }_{n\rightarrow \infty }\,b_{n}^{-1}\sum _{k=1}^{n}{\textsf{a}}_{k}^{\prime } =\infty$ a.e. since ${\textsf{a}}_{n}^{\prime }\le \sum _{k=1}^{n}{\textsf{a}} _{k}^{\prime }$. For the converse, assume that $\sum _{n\ge 1}1/(b_{n}\log b_{n})<\infty$, which by Lemma 2.1 entails $(n \log _2 n)/b_n \rightarrow 0$. In view of Theorem 2.1 a), our assumption implies that ${\textsf{M}}_{n}^{\prime }/b_n \rightarrow 0$ a.e. Together with statement b) above, these observations prove (2.7), because

$$\begin{aligned} \frac{1}{b_{n}}\sum _{k=1}^{n}{\textsf{a}}_{k}^{\prime }= \frac{{\textsf{M}}_{n}^{\prime }}{b_{n}}+ \frac{n\log _{2}n}{\log 2\cdot b_{n}}\cdot \frac{\log 2}{n\log _{2}n} \left( \sum _{k=1}^{n}{\textsf{a}}_{k}^{\prime } - {\textsf{M}}_{n}^{\prime } \right) \text {.} \end{aligned}$$

**d) ** Note first that letting $c_{n}^{\prime }:=d_{n}^{\prime }/\log _{2}(10j)$ for $n\in ({\overline{n}}(j-1),{\overline{n}}(j)]$, provides us with a non-decreasing sequence satisfying $\sum _{n\ge 1}1/(c_{n}^{\prime }\log c_{n}^{\prime })<\infty$ (use generous estimates). By Theorem 2.1 therefore $\lambda (\{{\textsf{M}}_{n}^{\prime }>c_{n}^{\prime }$ i.o.$\})=0$. Since $c_{n}^{\prime }=o(d_{n}^{\prime })$, we see that for every $\varepsilon >0$, $\{{\textsf{M}}_{n}^{\prime }>\varepsilon \,d_{n}^{\prime }$ i.o.$\}\subseteq \{{\textsf{M}}_{n}^{\prime }>c_{n}^{\prime }$ i.o.$\}$. Combining these observations shows that

5.4 $$\begin{aligned} \frac{{\textsf{M}}_{n}^{\prime }}{d_{n}^{\prime }}\longrightarrow 0\text { a.e.} \end{aligned}$$

Together with (2.5) and $n\log _{ 2} n/(\log 2\cdot d_{n}^{\prime })\le 1$, this proves, via

5.5 $$\begin{aligned} \frac{1}{d_{n}^{\prime }}\sum _{k=1}^{n}{\textsf{a}}_{k}^{\prime }=\frac{n\log _{2}n}{\log 2\cdot d_{n}^{\prime }}\cdot \frac{\log 2}{n\log _{2}n}\left( \sum _{k=1}^{n}{\textsf{a}}_{k}^{\prime }-{\textsf{M}}_{n}^{\prime }\right) +\frac{{\textsf{M}}_{n}^{\prime }}{d_{n}^{\prime }}\text {,} \end{aligned}$$

that

$$\begin{aligned} \underset{n\rightarrow \infty }{{\overline{\lim }}}\,\frac{1}{d_{n}^{\prime }} \sum _{k=1}^{n}{\textsf{a}}_{k}^{\prime }\le 1\text { a.e.} \end{aligned}$$

Specializing (5.5), and using (2.5) and (5.4) again, we find that

$$\begin{aligned} \frac{1}{d_{{\overline{n}}(j)}^{\prime }}\sum _{k=1}^{{\overline{n}}(j)} {\textsf{a}}_{k}^{\prime }=\frac{\log 2}{{\overline{n}}(j)\log _{2}{\overline{n}} (j)}\left( \sum _{k=1}^{{\overline{n}}(j)}{\textsf{a}}_{k}^{\prime }-{\textsf{M}} _{{\overline{n}}(j)}^{\prime }\right) +\frac{{\textsf{M}}_{{\overline{n}} (j)}^{\prime }}{d_{{\overline{n}}(j)}^{\prime }}\longrightarrow 1\text { a.e.} \end{aligned}$$

as $j\rightarrow \infty$, and our claim (2.8) follows. $\square$

### Proof of Theorem 2.3

**a)** Apply Theorem 4 of [3] to the system $(I,{\mathcal {B}}_{I},\mu _{{\mathfrak {G}}},S)$ with CF-mixing partition $\gamma :=\xi$. Statement (2.9) is immediate if we take $({\textsf{a}}^{\prime },{\textsf{a}}^{\prime })$ as our pair $(\varphi ,\psi )$ of $\gamma$-measurable functions, cf. Remark 3 in [3]. Turning to the general version (2.10), we consider $\varphi :=g\circ {\textsf{a}}^{\prime }$ and $\psi :={\textsf{a}}^{\prime }$. According to the result cited,

$$\begin{aligned} \underset{n\rightarrow \infty }{{\overline{\lim }}}\,\frac{g({\textsf{a}}_{n} ^{\prime })}{\sum _{k=1}^{n-1}{\textsf{a}}_{k}^{\prime }}=\infty \text { a.e. iff }\int _{I}a^{\prime }\circ g\circ {\textsf{a}}^{\prime }\,d\mu _{{\mathfrak {G}}}=\infty \text {, } \end{aligned}$$

(with $a^{\prime }$ from Lemma 4.1), while otherwise $\lim _{n\rightarrow \infty }\,g({\textsf{a}}_{n}^{\prime })/(\sum _{k=1} ^{n-1}{\textsf{a}}_{k}^{\prime })=0$ a.e. The present assertion merely reformulates the divergence condition above: We see (using (4.4) and the regularity properties on *g*) that (for some constant $c>0$)

$$\begin{aligned} \int _{I}a\circ g\circ {\textsf{a}}^{\prime }\,d\mu _{{\mathfrak {G}}}&\asymp \sum _{n\ge 1}\frac{g(\textrm{p}_{n})}{\log _{2}g(\textrm{p}_{n})} \,\mu _{{\mathfrak {G}}}(I_{\textrm{p}_{n}})\asymp \sum _{n\ge 1}\frac{g(n\log n)}{\log _{2}g(n\log n)}\frac{1}{(n\log n)^{2}}\\&\asymp \int _{c}^{\infty }\frac{g(x\log x)}{\log _{2}g(x\log x)}\frac{dx}{(x\log x)^{2}}\asymp \int _{c}^{\infty }\frac{g(y)}{\log _{2}g(y)}\frac{dy}{y^{2}\log y}\text {.} \end{aligned}$$

**b)** Same argument as in a), this time with $\varphi :=g\circ {\textsf{a}}^{\prime }$ and $\psi :={\textsf{a}}$, and replacing $a^{\prime }$ above by $a(t):=t/L(t)\sim \log 2\cdot t/\log t$ as $t\rightarrow \infty$.

**c)** We have

$$\begin{aligned} \lim _{n\rightarrow \infty }\frac{\sum _{k=1}^n {\textsf{a}}_k^{\prime }}{\sum _{k=1}^n {\textsf{a}}_k}&\le \lim _{n\rightarrow \infty }\frac{\sum _{k=1}^n {\textsf{a}}_k^{\prime }-{\textsf{M}}_n^{\prime }}{\sum _{k=1}^n {\textsf{a}}_k-{\textsf{M}}_n}+\lim _{n\rightarrow \infty }\frac{{\textsf{M}}_n^{\prime }}{\sum _{k=1}^n {\textsf{a}}_k-{\textsf{M}}_n}\\&\le \lim _{n\rightarrow \infty }\frac{n\log _2 n\log 2}{n\log n\log 2}+\lim _{n\rightarrow \infty }\frac{n\log _2 n\log 2}{n\log n\log 2}=0 \end{aligned}$$

which follows by b) of Theorem 2.2 together with the Diamond-Vaaler trimmed law, $\log 2\left( \sum _{k=1}^{n}{\textsf{a}} _{k}-{\textsf{M}}_{n}\right) /(n\log n)\rightarrow 1$ a.e. and finally by a) of Corollary 2.1. $\square$

### Proof of Theorem 2.4

**a) ** We have that $\int ({\textsf{a}}^{\prime })^{\gamma }\,\textrm{d}\lambda <\infty$ and the statement follows by the ergodic theorem.

**b) ** We may apply [15, Theorem 1.7 & erratum]. That Property ${\mathfrak {C}}$ is fulfilled with the bounded variation norm $\Vert \cdot \Vert _{BV}$ is a standard result. For Property ${\mathfrak {D}}$, we notice that $\Vert {\textsf{a}}\cdot \mathbbm {1}_{\{{\textsf{a}}\le \ell \}}\Vert _{BV}\le 2\ell$ and $\Vert \mathbbm {1}_{\{{\textsf{a}}\le \ell \}}\Vert _{BV}\le 2$ implying that this property is fulfilled.

In order to calculate the norming sequence $(d_n)$ we notice that

$$\begin{aligned} \mu _{{\mathfrak {G}}}\left( \left( {\textsf{a}}^{\prime }\right) ^{\gamma }>n\right)&=\mu _{{\mathfrak {G}}}\left( {\textsf{a}}^{\prime }>n^{1/\gamma }\right) \\&\sim \frac{1}{\log 2\, n^{1/\gamma } \log n^{1/\gamma }} =\frac{\gamma }{\log 2\, n^{1/\gamma } \log n}=\frac{L(n)}{n^{1/\gamma }}, \end{aligned}$$

where $L(n)=\gamma /(\log 2 \log n)$ is a slowly varying function.

Using then [15, Theorem 1.7 & erratum] we obtain that (2.12) holds for $(b_n)$ fulfilling $b_n=o(n)$ and $\lim _{n\rightarrow \infty }b_n\log _2 n=\infty$ and for $(d_n)$ fulfilling

$$\begin{aligned} d_n\sim \frac{1/\gamma }{1-1/\gamma } n^{\gamma } b_n^{1-\gamma }\left( L^{-\gamma }\right) ^{\#}\left( \left( \frac{n}{b_n}\right) ^\gamma \right) , \end{aligned}$$

where $\ell ^\#$ denotes the de Bruijn conjugate of a slowly varying function $\ell$, see e.g. [6] for a precise definition. In our case $\left( L^{-\gamma }\right) ^{\#}(n)=\left( (\log n)^\gamma (\log 2)^{\gamma }/\gamma ^{\gamma }\right) ^{\#}\sim \gamma ^{\gamma }/\left( (\log n)^\gamma (\log 2)^{\gamma }\right)$. Hence,

$$\begin{aligned} d_n\sim \frac{\gamma ^{\gamma }}{(\gamma -1)(\log 2)^{\gamma }} n^{\gamma } b_n^{1-\gamma } \frac{1}{\left( \log \left( n/b_n\right) ^\gamma \right) ^{\gamma }} \sim \frac{1}{(\gamma -1)(\log 2)^{\gamma }}\cdot \frac{n^{\gamma }b_n^{1-\gamma }}{(\log n)^{\gamma }}, \end{aligned}$$

where the last assymptotic follows from the assumption $b_n=o(n^{1-\epsilon })$. $\square$

## Proofs of the results on distributional convergence

We are now ready for the proofs of our distributional convergence results.

### Proof of Theorem 3.1

In all cases we only need to check convergence in law w.r.t. $\mu _{{\mathfrak {G}}}$.

**a) ** This follows directly from Theorem 2.4.

**b) ** This statement follows directly by [1]. We use the expression for $L'(N)$ from (4.3) which is a slowly varying function. Since for $b_n=n\log _2 n/\log 2$ we have $nL(b_n)\sim b_n$ the statement follows.

**c) ** This follows from [17, Theorem 1.8]. The conditions on the system and the asymptotic of the norming sequence $(d_n)$ we have already considered in the proof of Theorem 2.4. $\square$

### Proof of Theorem 3.3

In each of the three statements it suffices to prove distributional convergence under the invariant measure $\mu _{{\mathfrak {G}}}$ (see Propositions 3.1 and 5.1 in [25]).

**a)** For $A\in {\mathcal {B}}_{I}$ with $\lambda (A)>0$, the * (first) hitting time* function of *A* under the Gauss map *S*, $\varphi _{A}:I\rightarrow \overline{{\mathbb {N}}}:=\{1,2,\ldots ,\infty \}$ is given by $\varphi _{A}(x):=\inf \{n\ge 1:S^{n}x\in A\}$, which is finite a.e. on *I*. Define $S_{A}x:=S^{\varphi _{A}(x)}x$ for a.e. $x\in I$, which gives the *first entrance map* $S_{A}:I\rightarrow A$. Letting $A_{l}^{\prime }:=\{{\textsf{a}}^{\prime }\ge l\}$, $l\ge 1$, we see that $\varphi _{l}^{\prime }=\varphi _{A_{l}^{\prime }}$ and, more generally, $\varphi _{l,i}^{\prime }=\varphi _{A_{l}^{\prime }}\circ S_{A_{l}^{\prime }}^{i-1}$ for $i\ge 1$. It is clear that $A_{l}^{\prime }$ is $\xi$-measurable, and according to Lemma 4.1, $\mu _{{\mathfrak {G}}}\left( A_{l}^{\prime }\right) \sim (\log 2\cdot l\log l)^{-1}$ as $l\rightarrow \infty$. Therefore, Theorem 10.2.a) of [25] immediately implies statement a).

**b)** This is a straightforward consequence of Theorem 10.2.b) in [25], because $\{{\textsf{a}}^{\prime }\ge l/\vartheta \}=A_{\left\lfloor l/\vartheta \right\rfloor }^{\prime }$ is $\xi$ -measurable and (4.2) entails $\mu _{{\mathfrak {G}}}\left( {\textsf{a}}^{\prime }\ge l/\vartheta \right) \sim \vartheta \,\mu _{ {\mathfrak {G}}}\left( {\textsf{a}}^{\prime }\ge l\right)$ as $l\rightarrow \infty$.

**c)** Let ${\mathbb {P}}(j):=\{\textrm{p}\in {\mathbb {P}}:\textrm{p}\equiv j$ (mod *m*)$\}$, then Dirichlet’s PNT for primes in residue classes (e.g. Theorem 4.4.4 of [14]) asserts that for each *j* relatively prime to *m*,

$$\begin{aligned} \#\left( {\mathbb {P}}(j)\cap \{2,\ldots ,N\}\right) \sim \frac{1}{\phi (m)} \frac{N}{\log N}\quad \text {as }N\rightarrow \infty \text {.} \end{aligned}$$

Via an easy argument parallel to the proof of (4.2), this shows that

$$\begin{aligned} \mu _{{\mathfrak {G}}}\left( A_{l}^{\prime }\cap \left\{ {\textsf{a}}^{\prime }\equiv j\text { (mod }m\text {)}\right\} \right) \sim \frac{1}{\phi (m)\log 2} \cdot \frac{1}{l\log l}\text { as }l\rightarrow \infty \text {,} \end{aligned}$$

and hence $\mu _{{\mathfrak {G}}}\left( A_{l}^{\prime }(j)\right) \sim \mu _{ {\mathfrak {G}}}\left( A_{l}^{\prime }\right) /\phi (m)$ with $A_{l}^{\prime }(j):=A_{l}^{\prime }\cap \left\{ {\textsf{a}}^{\prime }\equiv j\text { (mod }m \text {)}\right\}$ a $\xi$-measurable set. Another direct application of Theorem 10.2.b) in [25] then completes the proof of our theorem. $\square$

The result thus established essentially contains (3.1).

### Proof of Theorem 3.2

Theorem 3.3 a) contains the statement that $\mu _{{\mathfrak {G}} }\left( A_{l}^{\prime }\right) \varphi _{l,1}^{\prime }$ converges to a standard exponential law. Using the natural duality $\{{\textsf{M}} _{n}^{\prime }<l\}=\{\varphi _{l,1}^{\prime }\ge n\}$ this is easily seen to imply (3.1). $\square$

### Proof of Theorem 3.4

The result follows directly by [16, Theorem 3] by considering the sets $A_n=\{{\textsf{a}}_n\in {\mathbb {P}}\cap \Gamma _n\}$. The only thing to check is that

6.1 $$\begin{aligned} \sum _{n=1}^{\infty }\lambda (A_n)\cdot \lambda (A_n^c)=\infty . \end{aligned}$$

We note that $\sum _{n=1}^{\infty }\lambda (A_n)\cdot \lambda (A_n^c)\ge \sum _{n=1}^{\infty }\lambda (A_n)$ and thus for (A) (6.1) follows from the proof of Theorem 2.1 a). (B) corresponds to [16, Theorem 5A] and for (C) (6.1) follows from the proof of Theorem 2.1 c). $\square$
